# Loneliness and the Degree of Addiction to Shopping and Work among Polish Women: The Mediating Role of Depression

**DOI:** 10.3390/jcm11216288

**Published:** 2022-10-25

**Authors:** Kamila Rachubińska, Anna Maria Cybulska, Ewa Kupcewicz, Alina Jurewicz, Mariusz Panczyk, Aneta Cymbaluk-Płoska, Justyna Jurczak, Elżbieta Grochans

**Affiliations:** 1 Department of Nursing, Pomeranian Medical University in Szczecin, 71-210 Szczecin, Poland; 2 Department of Nursing, Collegium Medicum, University of Warmia and Mazury in Olsztyn, 14 C Zolnierska Street, 10-719 Olsztyn, Poland; 3 Department of Clinical Nursing, Pomeranian Medical University in Szczecin, 71-210 Szczecin, Poland; 4 Department of Education and Research in Health Sciences, Faculty of Health Science, Medical University of Warsaw, 00-581 Warsaw, Poland; 5 Department of Gynecological Surgery and Gynecological Oncology of Adults and Adolescents, Pomeranian Medical University, 70-111 Szczecin, Poland; 6 Department of Social Medicine and Public Health, Pomeranian Medical University in Szczecin, 71-210 Szczecin, Poland

**Keywords:** behavioral addiction, depression, loneliness, compulsive buying behavior, workaholism

## Abstract

(1) The aim of the research was to try to define the mediating role of depression in the relationship between addiction to shopping and work and loneliness, understood in terms of general loneliness among Polish women. (2) The study was conducted among 556 women. The research was carried out with the use of the diagnostic survey method, using the questionnaire technique: the De Jong Gierveld Loneliness Scale, the Purchasing Behavior Scale, the Work Addiction Risk Test, Beck Depression Inventory, and our own questionnaire. (3) Depression is a mediator in the relationship between the feeling of loneliness and the degree of addiction to shopping (β = −0.0246, z = −2.03, *p* = 0.043) and in the relationship between the feeling of loneliness and the degree of addiction to work (β = −0.0722, z = −4.002, *p* < 0.001). The direct impact of the feeling of loneliness on the degree of addiction to shopping (*p* = 0.237) and work (*p* = 0.576) is statistically insignificant. (4) Depression plays the role of a mediator between the feeling of loneliness and the degree of addiction to shopping and work. An increase in the level of depression increases the degree of addiction to shopping and work. The mediator’s participation lowers the loneliness feeling level.

## 1. Introduction

The rapid development of civilization is accompanied by many changes affecting human functioning. Technological development, wide access to positive stimuli, and the neglect of emotional control and self-awareness in the learning process make it difficult for individuals to control their behavior. Such a loss of control is associated with the development of addictions [1]. Therapists, doctors, and researchers more and more often encounter cases of compulsive behavior focused on a specific activity; apart from gambling or playing computer or internet games, more and more often the subjects of preoccupation are compulsive shopping, sexual activities, and work (workaholism) [2].

Human work changes with the progress of civilization. In addition to its economic and social function, it creates the area of our existence in which we are looking for opportunities for professional and personal development. Changes in the work process resulted from changes in the functioning of the Polish economy, i.e., teleworking, temporary work, or work performed under a mandate contract. Those changes increase job insecurity; increased competition in terms of reducing costs, producing goods, improving their quality and employee productivity, and automating the work processes. These activities lead to a further increase in job insecurity, thus to greater involvement of employees in the performance of their tasks. This situation may be caused by various reasons, including reducing the sense of job security, increase in the value of work caused by the persistence of a high unemployment rate in Poland for a long period or, finally, the tendency of employers to give high gratification to those employees who contribute to the realization of the company’s mission [2,3].

The term “workaholism” was proposed by a psychiatrist Wayna Oates in 1971 to describe compulsive behaviors and thoughts related to the performance of a professional job [4]. The phenomenon of work addiction is gaining more and more interest among psychologists and therapists due to the increasing scale of the problem and its negative consequences for the individual and their environment. The diagnostic criteria of compulsive behavior included in both the ICD and DSM classification can be applied to workaholism. The characteristics of these criteria largely describe the symptoms of work preoccupation and loss of control [5].

Research on the buying process has shown that shopping, often treated as a way of spending free time, is also a stimulus that provides short-term gratification [6]. However, making intensive and excessive purchases may consequently be detrimental and even destructive to the individual. Compulsive Buying Disorder has been classified as Impulse-Control Disorders, not elsewhere classified in the DSM-IV classification. In the DSM-5 classification, compulsive buying disorder is described in the context of behavioral addiction, such as an exercise or sex addiction, but it has not been formally defined due to insufficient data [7].

Shopaholism and workaholism are addictions, as are alcoholism, drug addiction, gambling, sex addiction, and network addiction. They are based on the irresistible need to perform a specific activity [8]. The only difference from drug addiction or alcoholism is that there is no physiological dependence. Compulsive buying and shopaholism are a different type of addiction called additive buying. A shopaholic behaves like any addict, knows that he is doing wrong, but he cannot get over it. Buying becomes a necessity, a compulsory behavior [9,10]. Loneliness plays a key role in the occurrence of addiction to shopping and work.

De Jong Gierveld [11] defines loneliness as an unpleasant, unacceptable situation of lack [quality] of certain social relations experienced by an individual. Loneliness is subjective and negative. It is the result of a cognitive assessment of the quantity and quality of existing relationships and the standards that an individual has for the relationship [12]. The results of the study by Cacioppo et al. showed that loneliness was a predictor of an increase in the level of depression [13]. Compulsive buyers have an increased level of social anxiety, alienation, a lowered level of self-esteem [14,15], and a high level of loneliness [16].

The first and most common psychiatric comorbid disease to look for in compulsive buying is depression. McElroy, Keck et al. (1994) [17] found that among 20 patients with compulsive buying disorder, 19 meet the DSM-III-R criteria for lifelong diagnosis of a major mood disorder, most often bipolar disorder [18].

Few studies have looked at the mechanisms underlying the relationship between workaholism and psychological problems, such as depression. A study by Yang et al. [19] showed a positive relationship between workaholism and depression. Workaholism, like other emerging behavioral addictions, such as Internet (gaming) addiction, is not a substance addiction and its causes, consequences, and mechanisms have not been well studied. Therefore, more theoretical and empirical research on workaholism is justified.

Rogowska et al. [20] found that gender plays an important role as mediator between workaholism and depression. Therefore, we eliminated gender as a variable in this research by focusing on the female population only. Research indicates that biological, social, and psychological factors contribute to the gender differences in the incidence of depression, which is confirmed by other studies [21]. Interestingly, it was found that in childhood, boys and girls equally report experiencing depression. However, in adulthood, women are two to three times more likely than men to be diagnosed with depression [22,23,24]. Chuick et al. [25] claim that men experience depression differently than women, which suggests that women internalize their depression more often, while men externalize it more often [26,27,28,29,30,31,32].

The aim of the research was to try to define the mediating role of depression in the relationship between addiction to shopping and work and loneliness, understood in terms of general loneliness among Polish women.

## 2. Materials and Methods

### 2.1. Settings and Design

The study was conducted among 556 women. Before starting the project, the approval of the Bioethics Committee of the Pomeranian Medical University in Szczecin was obtained (Resolution No. KB-0012/518/12/16). The inclusion criteria for the study were: female gender, age > 18 years of age, place of residence beingWest Pomeranian Voivodeship, submission of informed written consent to participate in the study, completion of the provided set of questionnaires. The prepared sheets of the research tool were handed out by the author to women who were acquainted with the above-mentioned information and agreed to participate in the project. After agreeing to participate in the study, the respondents received a set of questionnaires. During the distribution of the questionnaires, the author answered all questions about the research and the prepared tools. The respondents could withdraw from the study at any time without giving any reason. The test was performed in about 15 min.

### 2.2. Research Instruments

The research was carried out with the use of the diagnostic survey method, using the questionnaire technique. In order to analyze the occurrence of behavioral addictions in adult women, three standardized and adapted to Polish conditions research tools and an own questionnaire were used.

The De Jong Gierveld Loneliness Scale (DJGLS) by J. De Jong Gierveld and F. Kamphuis, Polish adaptation by P. Grygiel et al. The scale for measuring the sense of loneliness. DJGLS is basically one-dimensional and measures a generalized sense of loneliness. It is a partially balanced tool, consisting of five positive items measuring satisfaction with interpersonal relationships and six negative items describing dissatisfaction with social contacts. The level of acceptance of individual statements was indicated by the respondents on a 5-point scale, from “definitely yes” to “definitely not”. After recoding the “negative” items, a higher total score indicates a more intense feeling of loneliness. The scale is characterized by a high level of reliability and homogeneity: Cronbach’s internal stability coefficient is 0.89, the value of the mean inter-positional correlation r = 0.42, and the H Loevinger homogeneity coefficient—0.47 [33,34].

The Scale of Shopping Behavior (SZZ) is a scale to determine the risk of shopaholism. The tool allows us to determine the overall result of purchasing behavior and its two factors, which are compulsion and lack of control, and reduction of tension and negative emotions. The scale consists of 16 items, rated on a five-point scale (from almost never—1 to almost always—5). The overall sum of the scores is between 16–80 points. The higher the score, the greater the tendency for shopaholism. A high tendency for shopaholism is evidenced by a result above 44, and a low tendency by result below 35 points. The results in the range of 35–44 points indicate a moderate tendency towards shopaholism. Internal compliance of SZZ was assessed using Cronbach’s alpha coefficient, which is 0.92 [35].

The Work Addiction Risk Test (WART) is a questionnaire that measures the symptoms of a workaholic’s behavior pattern. The tool consists of 25 items measuring the behavioral, cognitive, and emotional responses that are believed to constitute workaholism syndrome. Twenty-five statements are rated on a four-point scale for the frequency of symptoms of work addiction. The test person’s task is to indicate (by marking one of the four categorized answers) to what extent each of the statements relates to him. The questionnaire measures fully formed workaholism syndrome or job addiction risk depending on the score. The range of the scale is from 25 to 100 points. The result of the compulsion to work is indicated by a score above 56 points: a high score (67–100 points) is an indicator of being highly addicted, a medium score (57–66 points) represents a moderate work addiction. The indicator of the lack of addiction and the degree of risk of the addiction to work is a low score ranging from 25–56 points (the higher the score, the greater the likelihood of developing workaholism) [36,37,38].

The Beck Depression Inventory-BDI I-II is a questionnaire used for measuring the severity of depressive disorders, and was developed by Aaron Beck et al. The tool consists of 21 questions with four options to answer (attitude and symptoms). Each category describes a specific behavioral manifestation of depression. The level of depression is calculated by adding up the total number of points obtained. The results of the calculations were interpreted by referring to standardized divisions, 0–13-no depression or minimal symptoms of depression, 14–19-mild depression, 20–28-moderate depression, and 29–63-severe depression [39].

### 2.3. Participants

556 women participated in the study. The respondents accounted for 0.1% of the female population of the West Pomeranian Voivodeship in Poland. The mean age of the respondents was 34 years, and the median was 27 years. The largest group (40.3%) were women aged 20–30. Due to the small size of the selected subgroups, the analysis of socio-demographic variables (i.e., education and marital status) was based on the following classification criteria: education: higher and lower (secondary, vocational, primary), marital status: in a formal/informal relationship and single (maiden, divorced, widow).

Of the 556 respondents, 48.4% had higher education, and slightly more than half of them lower (51.6%). Women living in towns with less than 100,000 inhabitants accounted for over half of the respondents (52.3%). Most of the women declared staying in a formal/informal relationship (66.5%). The vast majority of the respondents, 89.2%, remained professionally active (Table 1).

### 2.4. Statistical Analysis

Quantitative data collected using standardized scales was presented using the parameters of descriptive statistics. The following were determined: mean, standard deviation (SD), range (min-max), and skewness. In order to obtain the distribution of symmetrical analyzed variables, they were transformed with the Box-Cox method [40]. A Box-Cox transformation is a transformation of non-normal variables into a normal shape. Normality is an important assumption for mediation analysis. The Generalized Linear Model Mediation Analysis was used to estimate the influence of the mediator (depressiveness) on the relationship between the independent variable and the dependent variable. A mediation model was fitted to each sample resulting in a bootstrap sample. Bootstrapping is a statistical method that utilizes random resampling with replacement to estimate a population parameter. This technique samples from a given dataset to estimate a parameter when it would otherwise be impossible [41].

Data were analyzed using IBM SPSS (Version 28, IBM Corp., Armonk, NY, USA) for the descriptive analyses, and Jamovi (Version 2.2.5, Jamovi Project, Sydney, Australia), with jAMM module to test the mediation model [42]. The jAMM package allows estimation of the direct and indirect effects of the independent variables on the dependent variables, by also examining all paths of the mediation model components (e.g., the associations between the independent variables and the mediator and the associations between the mediator and the dependent variables) [43]. For all analyses, a 5% level of statistical significance was set to reject the null hypothesis.

## 3. Results

Over the course of statistical analyses, mean values of the analyzed variables were established. The De Jong Gierveld Loneliness Scale measured a generalized feeling of loneliness. The mean value of the scores obtained by the respondents on the Sense of Loneliness Scale was 34.01 ± 3.87.

The mean scores obtained in the Scale of Shopping Behavior are 27.00 ± 10.77. The WART questionnaire measures the symptoms of a workaholic behavior pattern. The respondents achieved an average of 53.00 ± 12.24 points. Beck Depression Inventory-BDI I-II measures symptoms of depression. The mean value of the scores is 34.00 ± 3.87 (Table S1).

### Mediation Analysis (Generalized Linear Model Mediation Analysis, Model Parameters Estimated Using Bootstrap Method)

In order to check whether depression is a significant mediator of the relationship between loneliness (De Jong Gierveld) and the overall assessment of the degree of addiction to shopping (SZZ) and work (WART), a mediation analysis was carried out.

In the mediation model adopted, it has been shown that depression is a mediator between the feeling of loneliness and the degree of addiction to work and shopping. The increase in the level of depression increased the degree of addiction to shopping. The mediator’s participation lowered the level of the feeling of loneliness.

Model 1 (Figure 1).

The results of the conducted analysis revealed the existence of a statistically significant mediation. Depressiveness is a mediator in the relationship between the feeling of loneliness and the degree of addiction to shopping (β = −0.0246, z = −2.03, *p* = 0.043). The mediation effect accounts for 29.1% of the variability of the dependent variable.

The relationship between loneliness and depression is negative (β = −0.2252, z = −4.64, *p* < 0.001). The relationship between depression and addiction to shopping is positive (β = 0.1092, z = 2.42, *p* = 0.015). The increase in the level of depression increased the degree of addiction to shopping. The mediator’s participation lowered the level of the feeling of loneliness. Loneliness was not a significant predictor of shopping addiction. The direct impact of the feeling of loneliness on the degree of addiction to shopping is statistically insignificant (*p* = 0.237) (Table 2).

Model 2 (Figure 2).

The results of the conducted analysis revealed the existence of a statistically significant mediation. Depression is a mediator in the relationship between the feeling of loneliness and the degree of addiction to work (β = −0.0722, z = −4.002, *p* < 0.001). The mediation effect accounts for 75.1% of the variability of the dependent variable.

The relationship between the feeling of loneliness and depression is negative (β = −0.2252, z = −4.70, *p* < 0.001). The relationship between depression and work addiction is positive (β = 0.3207, z = −7.524, *p* < 0.001). The increase in the level of depression increased the degree of addiction to work. The mediator’s participation lowered the level of the feeling of loneliness. Loneliness was not a significant predictor of work addiction. The direct impact of the feeling of loneliness on the degree of addiction to work is statistically insignificant (*p* = 0.576) (Table 3).

## 4. Discussion

Therapists, doctors, and researchers more and more often encounter cases of compulsive behavior focused on a specific activity. Apart from gambling or playing computer or internet games, increasingly the subjects of preoccupation are compulsive shopping, sexual activities, and work (workaholism) [5].

To the best of our knowledge, this is the first study to investigate the mediating role of depression in the relationship between shopping, work addiction, and loneliness. The results obtained in this study provided the following contribution to the literature.

Research in the field of behavioral addictions has shown that people who over-engage in certain activities usually struggle with problematic social lives and often experience a lack of social support and a feeling of loneliness [44,45,46,47]. Not much is known regarding social functioning of people addicted to shopping compared to other addictions. Based on previous studies [48,49,50], it can be expected that addiction to shopping is also associated with loneliness. Compulsive buying can be a way to escape the feeling of alienation, but it can also contribute to the escalation of interpersonal conflicts through the constant intensification of behavior [44].

The average score on the SZZ scale was 30.44, which indicates a low risk of shopaholism. Mueller et al. [51] noted a prevalence of addiction to purchasing of 6.9%. Age was inversely proportional to the prevalence of compulsive buying. Addicts showed greater depression than non-addicts [51,52,53]. Otero-López and Villardefrancos [50] showed the occurrence of addiction to purchases with the frequency of 7.1%. Female gender, depression, anxiety, and younger age were predictors of compulsive buying. Most studies have reported higher prevalence rates in women than in men [18,51,52,54].

In the mediation model adopted, it has been shown that depression is a mediator between the feeling of loneliness and the degree of addiction to shopping. The increase in the level of depression increased the degree of addiction to shopping. The mediator’s participation lowered the level of the feeling of loneliness. Other studies have shown similar results [14,15]. Compulsive buyers showed an increased level of social anxiety, decreased self-esteem, and a high sense of loneliness [14,15,16]. Thus, it can be concluded that psychological factors (e.g., feeling of loneliness) may be an important predictor of compulsive buying.

Research by Uzarska et al. [44] showed a positive correlation between social anxiety and loneliness and addiction to shopping, which may result from the fact that people who engage in excessive spending have problems with maintaining close relationships. This is consistent with previous research on addiction to shopping [48,49,50], as well as with other addictions [46,55].

Lejoyeux et al. [27] conducted research on the prevalence of shopaholism among hospitalized patients who showed features of a severe depressive episode. The addicts most often were younger people, women, and people who were single. People displaying compulsive buying were characterized by a greater number of depressive symptoms assessed using the German version of the Short Health Mood Scale (PHQ-9) [51]. Black et al. [56] demonstrated in their research that compulsive buyers were accompanied by mood disorders, anxiety, depressive symptoms, or ADHD throughout their lives. The results of the research by Suresh et al. [57] showed that psychological factors, such as loneliness, depression, low self-esteem, and anxiety, were positively associated with Internet addiction and compulsive online buying.

The main findings revealed a clear mediation: it has been shown that depression is a mediator between the feeling of loneliness and the degree of addiction to work. The increase in the level of depression increased the degree of addiction to work. The mediator’s participation lowered the level of the feeling of loneliness. Loneliness was not a significant predictor of work addiction.

Depression is one of the most common causes of work disability in industrialized countries [58,59,60,61]. A Finnish study showed that 50% of men and 28% of women with the first episode of depression were diagnosed with OCPD/APD [62,63]. First, workaholism is associated with increased stress and a disturbed work-life balance. On the other hand, stress is positively associated with depression [64]. According to the study by Yang et al. [19], respondents for whom professional career was more important than maintaining work-life balance were characterized by a higher level of depression. This is in line with previous studies [65].

Mental disorders, such as anxiety and depression, may increase the risk of addiction [62,63]. They can lead to addiction and vice versa [64,65,66,67,68]. Many studies have previously reported an association between anxiety, depression, and workaholism [69,70,71,72,73,74,75,76]. In addition, it is known that workaholism (in some cases) may result from an attempt to reduce the unpleasant feeling of anxiety and depression. Hard work is praised and honored in modern society and thus serves as the legitimate behavior of individuals to combat or mitigate negative feelings, and to feel better and increase self-esteem [77,78].

The average score on the WART scale was 53.46 points; a score in the range of 25–56 points indicates a low risk of workaholism, but the higher the number of points, the greater the risk of addiction.

Work addiction is associated with higher levels of stress at work and outside [79], sleep disturbances [80,81,82] and decreased well-being [82,83,84,85]. In contrast, chronic stress is a well-recognized risk factor for major depression as well as for many other disorders and non-communicable diseases [79]. Similar results were obtained by other authors [66,83,84,86,87]. The importance of the relationship between work addiction and depression and loneliness gains a new perspective after taking into account the socioeconomic costs of chronic stress [74,87,88,89]. Andreassen et al., have shown in their research that workaholism has a significant influence on the mediation between work-related stress and health-related outcomes, including emotional exhaustion, somatic symptoms, social dysfunction, and insomnia [90].

Depression seems to be significantly related to workaholism, leading directly to work addiction (and vice versa) [68,91,92]. The relationship between workaholism and depression has been demonstrated in studies conducted in various professional and cultural contexts [66,69,70,93,94]. Haar and Roche [89] found that both work commitment and joy at work were associated with anxiety and depression. Houlfort et al. [94] found that depression was positively correlated with an obsessive passion for work. Similarly, Nie and Sun [70] found a significant correlation between workaholism and depression. It is worth mentioning that a cross-sectional survey on a large sample of 16,426 employees showed positive and significant correlations between workaholism and all the examined symptoms of mental disorders, including depression [66]. The results revealed a partial mediating role of burnout in the relationship between work addiction and depression. Hard work is valued in society. In this way, it can serve as justified and rationalized behavior of individuals in order to reduce negative feelings, feel better, and raise self-esteem [66]. Consequently, employers should be aware of the negative consequences of workaholism and understand that overworking does not equal productivity. Employers should adapt working conditions by establishing standards and values that ensure that both labor productivity and work-health balance are maintained, and by offering their employees training in time and stress management [81].

Workaholism, like other emerging behavioral addictions, such as Internet (gaming) addiction, is not a substance addiction, and its causes, consequences, and mechanisms have not been well studied. Therefore, further theoretical and empirical research on workaholism is justified [19].

These results provide empirical insight into the social functioning of people addicted to shopping and work. Loneliness and social anxiety with the mediating role of depression make it impossible for people to seek social support and deal with various problems. As a result, the lack of social support, along with the mediating role of depression may favor non-adaptive coping through compulsive shopping or workaholism. At the same time, addictions may cause conflicts with the social environment, which favors further isolation from relatives [44]. Therefore, it seems important to continue the research and conduct more detailed analysis of social predictors of addiction to shopping and work.

## 5. Limitations and Implications for Professional Practice

The divagations presented in this study on the role of depression as a mediator in the relationship between addiction to shopping, work, and loneliness among Polish women identified certain limitations and implications for professional practice. The main strengths of this study were the inclusion of important and reliable psychometric tools. Moreover, the presented data was enriched by using open-ended questions in addition to standardized tools. Consequently, the study significantly enriches the existing literature on behavioral addictions and provides further insight into the nature of shopping and work addiction, and its relationship to mental health and well-being. The presented research also has its limitations, including the sample size. The Polish sample was not representative, which places restrictions on the possibility of generalization to other populations and the exclusion of the male population. Since the cross-sectional research design does not allow for causal inference, it is necessary to design a causal experiment to fully clarify the role of the independent variables and the mediator.

The model did not test other potentially important predictors of behavioral addiction development, i.e., anxiety, stress, and insomnia. Despite the limitations, this study provides important findings and may be a starting point for wider research on the impact of psychological factors on the occurrence of addictions to shopping and work among Polish women. From a theoretical point of view, our findings are important as they provide additional insight into the relationship between workaholism and depression. Mental health professionals should be aware of the relationship between depression, workaholism, and shopaholism, and gender, in order to implement appropriate preventive programs and accurately select the target group of therapy.

## 6. Conclusions

In the mediation model adopted, it was shown that depressiveness plays the role of a mediator between the feeling of loneliness and the degree of addiction to shopping and work. The increase in the level of depression increased the degree of addiction to shopping and work. The mediator’s participation lowered the level of the feeling of loneliness. Loneliness was not a significant predictor of addiction to shopping and work. It is noteworthy that there are other variables (such as marital/romantic satisfaction, quality of friendship, hobbies/leisure time, impulsivity, emotional regulation) that could increase the explanatory capacity of the model in future studies. There is a need to include activities aimed at identifying psychological factors influencing the occurrence of addictions to shopping and work among women. It seems important to be able to use psychological help when needed. It is also necessary to take institutional preventive measures to prevent the occurrence of behavioral addictions among women.

## Figures and Tables

**Figure 1:**
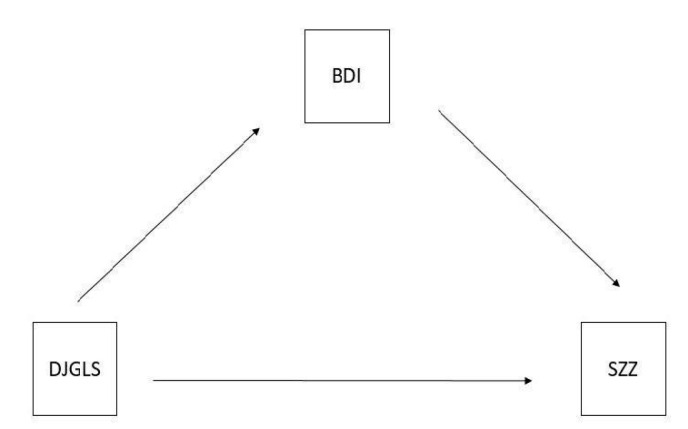
Mediation model 1. DJGLS-De Jong Gierveld Loneliness Scale, SZZ-Scale of Shopping Behaviour, BDI-Beck Depression Inventory–BDI I-II.

**Figure 2:**
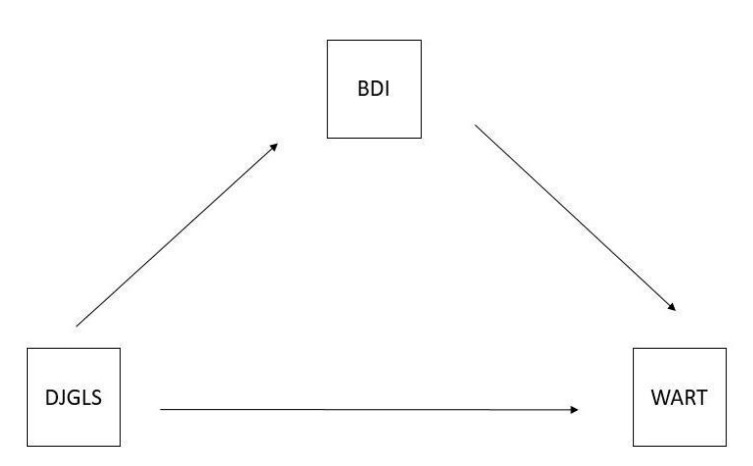
Mediation model 2 DJGLS-De Jong Gierveld Loneliness Scale, WART-Work Addiction Risk Test, BDI-Beck Depression Inventory–BDI I-II.

**Table 1:** General sociodemographic characteristics of the study group (n = 556).

Sociodemographic Variables		n	%
Education	Lower (secondary, vocational, elementary)	287	51.6
	Higher	269	48.4
Marital status	Single (maiden, divorced, widow)	186	34.5
	in a formal/informal relationship	370	66.5
Place of residence	<100.000 inhabitants	293	52.7
	≥100.000 inhabitants	263	47.3
Professional activity	Professionally active	496	89.2
	Professionally inactive	60	10.8

n-number of cases, %-percentage of the total study group.

**Table 2:** Indirect and Total Effects-mediation model 1.

Type	Effect	Estimate	95% C.I. Lower	95% C.I. Upper	β	Z	*p*
Indirect	DJGLS -> BDI -> SZZ	−4.62 × 10^−6^	−9.02 × 10^−6^	−8.06 × 10^−8^	−0.0246	−2.03	0.043
Component	DJGLS -> BDI -> SZZ	−8.62 × 10^−4^	−0.00123	−5.03 × 10^−4^	−0.2252	−4.64	<0.001
		0.00536	9.0006 × 10^4^	0.00958	0.1092	2.42	0.015
Direct	DJGLS -> SZZ	−1.12 × 10^−5^	−2.97 × 10^−5^	7.57 × 10^−6^	−0.0598	−1.18	0.237
Total	DJGLS -> SZZ	−1.59 × 10^−5^	−3.14 × 10^−5^	−2.76 × 10^−7^	−0.0844	−1.99	0.046

DJGLS-De Jong Gierveld Loneliness Scale, SZZ-Scale of Shopping Behaviour, WART-Work Addiction Risk Test, BDI-Beck Depression Inventory–BDI I-II, *p*—significance level, β—regression coefficient. Note. Confidence intervals computed with method: Parametric bootstrap, betas are completely standardized effect sizes.

**Table 3:** Indirect and Total Effects-Mediation model 2.

Type	Effect	Estimate	95% C.I. (a) Lower	95% C.I. (a) Upper	β	Z	*p*
Indirect	DJGLS -> BDI -> WART	−7.51 × 10^−4^	−0.00110	−3.67 × 10^−4^	−0.0722	−4.002	<0.001
Component	DJGLS -> BDI -> WART	−8.62 × 10^−4^ 0.871	−0.00121 0.64018	−4.88 × 10^−4^ 1.09	−0.2252 0.3207	−4.700 7.524	<0.001 <0.001
Direct	DJGLS -> WART	−2.48 × 10^−4^	−0.00108	6.59 × 10^−4^	−0.0239	−0.559	0.576
Total	DJGLS -> WART	−10.00 × 10^−4^	−0.00186	−1.38 × 10^−4^	−0.0961	−2.274	0.023

DJGLS-De Jong Gierveld Loneliness Scale, SZZ-Scale of Shopping Behavior, WART-Work Addiction Risk Test, BDI-Beck Depression Inventory–BDI I-II, β—regression coefficient. Note. Confidence intervals computed with method: Parametric bootstrap, Betas are completely standardized effect sizes, *p*—significance level.

## Data Availability

The data presented in this study are available on request from the first author.

## Supplementary Materials

The following supporting information can be downloaded at: https://www.mdpi.com/article/10.3390/jcm11216288/s1, Table S1. Description of primary variables and after Box-Cox transformation for N = 55.
