# Supplementary material for: Loneliness and the Degree of Addiction to Shopping and Work among Polish Women: The Mediating Role of Depression
Source: J Clin Med. 2022 Oct 25;11(21):6288. doi: 10.3390/jcm11216288 (PMC9654372; doi:10.3390/jcm11216288)
Supplement: Supplementary file 1 [file jcm-11-06288-s001.zip › jcm-1934162-supplementary.pdf]

Table S1. Description of primary variables and after Box-Cox transformation for N = 55

Variables	Shapiro-Wilk	M	SD	Me	Q1 - Q3	Min - Max
DJGLS	<0.00001	34.00	3.870	34.01	32.00 - 36.00	13.00 - 45.00
DJGLS after Box-Cox transformation	0.00001	1232	305	1255	1075 - 1401	142 - 2313
SZZ	<0.00001	27.00	10.77	30.44	22.00 - 38.00	16.00 - 71.00
SZZ after Box-Cox transformation	<0.00001	1.578	0.0572	1.584	1.539 - 1.634	1.469 - 1.715
WART	0.00538	53.00	12.24	53.46	45.00 - 62.00	25.00 - 95.00
WART after Box-Cox transformation	0.0820	19.26	3.169	19.25	17.14 - 21.52	11.14 - 29.01
BDI	<0.00001	4.500	7.345	6.800	1.00 - 10.00	0 - 40.00
BDI after Box-Cox transformation	<0.00001	1.843	1.166	1.783	0.718 - 2.686	0 - 4.433

M – mean, Me – median, SD – standard deviation, Min – minimum, Max – maksimum, Q1 – lower quartile, Q3 – upper quartile, DJGLS - De Jong Gierveld Loneliness Scale, SZZ - Scale of Shopping Behaviour , WART - Work Addiction Risk Test , BDI - Beck Depression Inventory – BDI I-II
